# Differential Effects of Experience and Information Cues on Metacognitive Judgments About Others’ Change Detection Abilities

**DOI:** 10.1177/20416695211039242

**Published:** 2021-08-26

**Authors:** Jeniffer Ortega, Patricia Montañes, Anthony Barnhart, Gustav Kuhn

**Affiliations:** Departamento de Psicologia, Universidad Nacional de Colombia, Bogota, Colombia; Departamento de Psicologia, Universidad El Bosque, Bogota, Colombia; Departamento de Psicologia, Universidad Nacional de Colombia, Bogota, Colombia; Department of Psychological Science, Carthage College, Kenosha, Wisconsin, United States; Department of Psychology, Goldsmiths University of London, London, United Kingdom

**Keywords:** visual metacognition, change blindness, information cues, experience cues, System 1, System 2, metacognitive bias, magic

## Abstract

This study explored the interaction between visual metacognitive judgments about others and cues related to the workings of System 1 and System 2. We examined how intrinsic cues (i.e., saliency of a visual change) and experience cues (i.e., detection/blindness) affect people’s predictions about others’ change detection abilities. In Experiment 1, 60 participants were instructed to notice a subtle and a salient visual change in a magic trick that exploits change blindness, after which they estimated the probability that others would detect the change. In Experiment 2, 80 participants watched either the subtle or the salient version of the trick and they were asked to provide predictions for the experienced change. In Experiment 1, participants predicted that others would detect the salient change more easily than the subtle change, which was consistent with the actual detection reported in Experiment 2. In Experiment 2, participants’ personal experience (i.e., whether they detected the change) biased their predictions. Moreover, there was a significant difference between their predictions and offline predictions from Experiment 1. Interestingly, change blindness led to lower predictions. These findings point to joint contributions of experience and information cues on metacognitive judgments about other people’s change detection abilities.

For centuries magicians have astounded people with illusions of impossibility. Although spectators often praise magicians for their sleight of hand and skillful deception, it is our brains that hold the keys to these illusions. Most magic tricks are underpinned by perceptual and cognitive failures. For instance, empirical research shows that we often miss unexpected salient visual stimuli (inattentional blindness) or visual changes to our environment (change blindness) (Rensink, 2009). Our brains are riddled with perceptual and cognitive failures and yet most people are surprised by the ease by which magicians can prevent their audience from perceiving salient stimuli in their environment. Several studies have shown that we are typically unaware of our perceptual limitations which results in biased and inaccurate judgments about our mental abilities (Levin et al., 2000; Ortega et al., 2018). Therefore, surprise is linked to another built-in feature: metacognitive failures. In other words, lack of knowledge about our cognitive blindspots may lead us to overestimate or underestimate our mental capabilities.

Metacognitive judgments can be supported by two distinct cognitive systems (Arango-Muñoz, 2010). According to Kahneman (2011), System 1 “operates automatically and quickly, with little or no effort and no sense of voluntary control” (p. 20), whereas System 2 “allocates attention to the effortful mental activities that demand it, including complex computations” (p. 21). Based on the operations of these two systems, Koriat et al. (2008) have argued that metacognitive judgments might be either information-based or experience-based.

Different cues underpin both types of metacognitive judgments: the person’s beliefs about how the mind works (folk theories), beliefs about the characteristics of the stimuli (intrinsic cues), beliefs about the conditions in which the stimuli are presented (extrinsic cues), or feelings derived from online task performance (mnemonic cues) (Koriat, 1997; Koriat & Ackerman, 2010; Koriat et al., 2008). The operations of System 1 give rise to experience-based metacognitive judgments or intuitive decisions devoid of declarative content. In contrast, the operations of System 2 give rise to information-based metacognitive judgments that result from conscious analytical thinking and the use of working memory to make comparisons and construct mental models.

The accuracy of metacognitive judgments depends on the validity of underlying beliefs and feelings (Koriat & Bjork, 2006). For instance, people believe that large words will be easier to remember than small words (Rhodes & Castel, 2008; Su et al., 2018), or that loud words will be easier to remember than quiet words (Frank & Kuhlmann, 2017). Contrary to participants’ beliefs, the characteristics of the stimuli (information cues) do not seem to affect memory performance. Several studies have found that people believe that they are better at noticing changes that usually go unnoticed (a phenomenon known as *change blindness blindness*), and sometimes they also show underconfidence in visual tasks (Beck et al., 2007; Levin, 2002; Levin & Angelone, 2008; Levin et al., 2002; Levin et al., 2000). In a change blindness blindness study (Beck et al., 2007), participants were better at detecting changes when they intentionally looked for them, than when they did not look for them. When other participants were asked to predict performance, they judged both conditions (i.e., incidental and intentional) to be equally difficult. This finding illustrates that observers are often unaware of how intention affects change detection.

Other studies have demonstrated how experiential cues impact metacognitive judgments. For instance, Kelley and Jacoby (1996) found that the time subjects spent solving anagrams correlated with their judgment of how difficult it would be for others to solve them. Koriat and Ackerman (2010) found that when participants performed a self-paced study task, they used a memorizing-effort heuristic to make metacognitive judgments about others—the more time they spent studying an item, the lower their predicted likelihood of recalling it later. However, when participants made judgments about others without having done the self-paced study task themselves, they failed to apply the memorizing-effort heuristic and instead based their judgements on the belief that recall should increase with study time. Loussouarn et al. (2011) found that when participants had to make predictions about their own change detection ability, their metacognitive judgments were based on an effort heuristic. That is, when they spent more time looking for a change, they were less confident in their change detection ability. In contrast, when they spent less time looking for the change, they were more confident.

Metacognitive judgments about our perceptual and cognitive abilities are consistently biased by both information and experience cues. In common life situations we often need to estimate other people’s perceptual abilities (e.g., software design, assessment of eyewitness accuracy, display design). Understanding the mechanisms that underpin our judgements about ourselves and others has important practical and theoretical implications, and yet, we do not know whether these judgments are susceptible to the same biases and errors. If change blindness blindness is a case of illusory superiority, perhaps judgments about others would be less biased and optimistic. However, people also overestimate others’ change detection abilities (Levin et al., 2000). Furthermore, one of our previous studies (Ortega et al., 2018) found that when participants detected an unexpected visual change in a magic trick, they judged that others would be more likely to notice it. On the other hand, those who missed the visual change made lower estimates. It is therefore likely that people’s own experience influences their metacognitive judgments about others. This raises questions about the interaction between visual metacognitive judgments about others and different types of cues (i.e., information and experience). For instance, will the experience of change blindness yield significantly different predictions compared to predictions based on implicit beliefs about change detection? System 1 influences the decisions and explicit beliefs of System 2 through feelings and impressions, and given the characteristic “laziness” of System 2, the influence of System 1 is very powerful (Kahneman, 2011). Thus, our visual experiences could generate feelings and intuitions that, if endorsed by System 2, would turn into beliefs. However, System 1 is prone to biases and errors in specific circumstances.

In the present study, we set out to expand our previous research by exploring how the two systems may contribute differently to visual metacognition. We examined the following hypotheses: 1. Offline predictions (attributive) will differ as a function of the intrinsic cue (i.e., type of change: subtle and salient). 2. A salient visual change will be more easily detected than a subtle visual change. 3. Online predictions (retrospective) will differ as a function of the experience cue (i.e., detection or blindness). 4. Offline predictions will differ from online predictions. 5. First-hand experience with the visual change task will transfer to predictions for a nonexperienced change.

We used a magic trick that exploits change blindness. Magic tricks have become popular and useful tools in cognitive and neuroscientific research (Kuhn, 2019; Rensink & Kuhn, 2015). They have been used to study visual attention (Barnhart et al., 2019; Demacheva et al., 2012), the relationship between gaze and visual attention (Barnhart & Goldinger, 2014; Kuhn et al., 2008; Kuhn & Findlay, 2009; Kuhn & Tatler, 2005), the neural correlates of causality violations (Danek et al., 2015; Parris et al., 2009), the subjective feeling of free choice (Olson et al., 2015; Pailhès & Kuhn, 2021; Shalom et al., 2013), problem solving (Danek et al., 2014), and the influence of social cues in perception (Cui et al., 2011; Kuhn & Land, 2006) and visual metacognition (Ortega et al., 2018).

We used two versions of the Princess Card trick (credited to Henry Hardin, cf. Downs, 1909) in which the magician secretly changes the identity of five playing cards, a change that typically goes unnoticed (Ortega et al., 2018). In Experiment 1, we asked participants to notice the subtle and salient changes and predict the probability that others would notice each change. In Experiment 2, one group was asked to watch a version of the trick in which the change is subtle, and the other group was asked to watch the trick with a more salient change. Participants then had to estimate the probability that other people would notice the change. Subsequently, another saliency condition was described to them and participants had to make predictions for whether a third party would detect that change.

## Experiment 1

The aim of the first experiment was to induce participants to consult their prior beliefs about change detection by presenting two types of changes (i.e., subtle and salient) and examine whether this comparative mode of processing would result in differing predictions about others’ change detection abilities (Hypothesis 1).

### Method

#### Participants

Based on the findings of Ortega et al. (2018), the sample size was established assuming a power of .95 and an effect size of .4 resulting in a required sample of 24 participants. Anticipating data exclusions, we recruited 60 undergraduate students at Universidad El Bosque (mean age 32.8 ± 15.8 years; 43 females) to participate in the study in exchange for partial fulfillment of a class requirement. All participants had normal or corrected-to-normal vision, were naïve about the aim of the study and gave written informed consent.

#### Materials and Procedure

**Figure 1.**
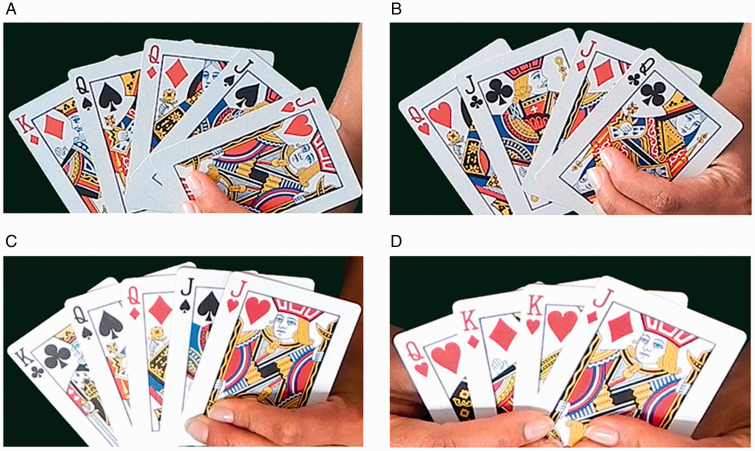
Subtle and salient changes in the princess card trick. *Note.* Subtle change: the cards presented at the beginning of the trick (A) have a different suit than the cards presented at the end of the trick (B) while the values and colors remain the same. Salient change: the cards presented at the beginning of the trick (C) are black and red, while the cards presented at the end are all red (D).

The experiment was conducted online. We used the Princess Card trick in which the magician makes a thought-of card disappear by secretly changing the identities of all the cards presented to the spectator. In our version of the trick (the second method described by Downs, 1909), the magician (first author) displays five cards in a fan and asks the spectator to think of one. Afterwards, the magician shuffles the cards and says that she can make the thought-of card disappear. She pretends to vanish the selected card with a hand movement. At the end, she counts only four cards and shows that the chosen card has vanished. To accomplish this trick, the magician uses trick cards that have different suits on the top and bottom corners. By manipulating the trick cards, the magician ensures that the chosen card no longer appears to be present. We manipulated the saliency of the change, thus creating two conditions. Empirical research has shown that short-term visual memory for a change that binds two or more features requires more attention and is more difficult than short-term visual memory for a change of one feature (Wheeler & Treisman, 2002). Thus, in the subtle condition, the same card values were presented after the change; however, their mappings onto card suits changed. For example, the jack of spades changed to the jack of clubs (Figure 1A and B) (supplementary video S1). In the salient condition, the cards presented at the beginning were black and red, while those presented at the end were all red (Figure 1C and 1D) (supplementary video S2). This type of change is easier to detect because it is based on one feature (i.e., the color of the cards) and does not require binding. Both versions of the trick were filmed at 29 fps using a Nikon COOLPIX L830 digital camera. The videos were edited in MAGIX Movie Edit Pro 2014 Plus and had durations of 47 seconds and 45 seconds.

Participants read the description (see Appendix A) of the subtle and salient versions of the Princess Card trick in counterbalanced order. Two figures were displayed to show the change (Figure 1). Afterwards, participants watched the video of each trick version and were then asked to estimate how many people out of 10 would notice the subtle and the salient change (0–10 scale).

### Results

#### Offline Metacognitive Judgments as a Function of the Intrinsic Cue

A mixed model ANOVA with between-subjects factor Order (subtle first, salient first) and within-subject factor Change Type (subtle, salient) showed a significant difference between metacognitive judgments for the subtle change (*M* = 4.13, *SD* = 2.2) and for the salient change (*M* = 5.1, *SD* = 2.6), *F*(1, 58) = 10.64, *p* = .002, η_p_^2^ = .16. The main effect of Order was nonsignificant, *F*(1, 58) = 2.15, *p* = .15, as was the interaction between Change Type and Order *F*(1, 58) = .8, *p* = .38. This result indicates that participants believed that others would detect the salient change more easily than the subtle change. Four remaining hypotheses were addressed in the next experiment.

## Experiment 2

The aim of this experiment was to examine differences in detection rates between the subtle and the salient visual change, online predictions as a function of the experience cue, and differences between offline and online predictions (Hypothesis 2–4). Moreover, we examined whether the first-hand experience with the visual change task transfers to metacognitive judgments for a nonexperienced change (Hypothesis 5).

### Method

#### Participants

Based on the findings of Ortega et al. (2018), the sample size was established assuming a power of .95 and an effect size of .4 resulting in a required sample of 84 participants. Ninety-five undergraduate students at the Universidad Nacional de Colombia participated in the study in exchange for partial fulfillment of a class requirement. All participants had normal or corrected-to-normal vision, were naïve about the aim of the study, and gave written informed consent. We excluded participants who had seen the trick before (12) or had incomplete questionnaires (3), resulting in a sample of 80 (mean age 21.6 ± 3.1 years; 50 females).

#### Materials and Procedure

Participants were randomly assigned to the subtle (*n* = 40) or the salient condition (*n* = 40). After watching the video clip, participants were asked to complete a questionnaire (see Appendix B). On the first page, subjects had to report whether they had seen the magic trick before and whether they noticed anything unusual about the trick. If subjects answered yes to the latter question, they were asked to describe what they saw. On the second page, participants were presented with two figures that showed the change of the cards, and asked whether they had noticed that the cards presented at the end of the trick were different from the cards presented at the beginning. Subjects were then asked to estimate how many people out of 10 would notice the change (0–10 scale). Finally, participants were shown two figures corresponding to a nonexperienced change: Namely, the subtle group had to imagine that the cards changed as shown in Figure 1C and D (salient change), and the salient group that the cards changed as shown in Figure 1A and B (subtle change). They were instructed to predict how many people out of 10 would detect the change.

### Results

#### Detection Rates and Saliency Manipulation

Participants were classified as having detected the change if they reported noticing it. In total, 22.5% of the participants noticed the subtle change, while 72.5% detected the salient change. As expected, the salient change was more easily detected than the subtle one, χ^2^ (1, *n* = 80) = 20.05, *p* < .001.

#### Online Predictions as a Function of the Experience Cue

Our first analysis focused on participants’ estimates of the probability that others would notice a change that they themselves had observed or missed. A two-way ANOVA with Experience Cue (change blindness, change detection) and Intrinsic Cue (subtle change, salient change) as between-subjects factors produced a significant main effect of Experience Cue, *F*(1, 76) = 31.67, *p* < .001, η_p_^2^ = .294. Participants who detected the change provided significantly higher predictions (*M* = 5.71, *SD* = 2.07) than those who missed it (*M* = 2.95, *SD* = 1.58). In contrast, the main effect of Intrinsic Cue was nonsignificant, *F*(1, 76) = .184, *p* = .67, as was the interaction between Experience Cue and Intrinsic Cue, *F*(1, 76) = 3.49, *p* = .065.

#### Online and Offline Predictions for the Subtle and the Salient Change

This analysis examined differences between offline predictions (Experiment 1) and online predictions (Experiment 2) for each type of change. After checking the assumptions for one-way ANOVA, a Welch test was conducted for predictions on the subtle change and multiple comparisons were done through the Games-Howell procedure. There was a significant difference between offline and online predictions, *F*(2, 21.5) = 4.1, *p* = .031. The Games-Howell *post hoc* test revealed that participants with change blindness predicted significantly less detection (*M* = 3.13, *SD* = 1.61) than participants who made the predictions offline (*M* = 4.13, *SD* = 2.24) (*p* = .042), whereas the online predictions in the change detection group were similar to offline predictions (*p* = .65). Similarly, a Welch test revealed a significant difference between offline and online predictions for the salient change, *F*(2, 34.1) = 19.71, *p* < .001. The Games-Howell *post hoc* test revealed that participants with change blindness predicted significantly less detection (*M* = 2.45, *SD* = 1.44) than participants who made the predictions offline (*M* = 5.1, *SD* = 2.61) (*p* < .001), whereas the online predictions in the change detection group were similar to offline predictions (*p* = .16).

#### Estimated Detection Rates for the Non-Experienced Change

We examined whether prior detection experiences influenced participants’ predictions for a nonexperienced change of differing saliency. A two-way ANOVA with Experience Cue (change blindness, change detection) and Intrinsic Cue (subtle change, salient change) as between-subjects factors found a significant main effect of Intrinsic Cue, *F*(1, 76) = 51.82, *p* < .001, η_p_^2^ = .405. Predicted detection rates were significantly higher for the salient change (*M* = 7.6, *SD* = 2.27) than for the subtle change (*M* = 3.65, *SD* = 2.4). The main effect of Experience Cue was nonsignificant, *F*(1, 76) = 1.76, *p* = .19, as was the interaction between Experience Cue and Intrinsic Cue, *F*(1, 76) = .22, *p* = .64.

## Discussion

A dual-process perspective on metacognition proposes that metacognitive judgments are underpinned by System 1 and System 2, which implies that metacognitive judgments might be experience-based or information-based, respectively. Several studies have found that information-based judgments might lead to metacognitive errors when they are based on wrong beliefs (Frank & Kuhlmann, 2017; Levin et al., 2000; Rhodes & Castel, 2008; Su et al., 2018). Other studies have shown that experience-based judgments might generate egocentric biases (Kelley & Jacoby, 1996; Koriat & Ackerman, 2010; Ortega et al., 2018).

In the present study, we explored the interplay between visual metacognitive judgments about others and different types of cues (i.e., information and experience). For instance, will the experience of change blindness yield significantly different predictions compared to predictions based on implicit beliefs about change detection? Several ideas support the notion that people believe that they are better at detecting changes than they actually are (Levin et al., 2000). First, perception seems effortless and it works wonderfully for most daily life purposes without our awareness of how it works. As Hoffman (1998) puts it, “vision is normally so swift and sure, so dependable and informative, and apparently so effortless that we naturally assume that it is, indeed, effortless” (p. xi). Second, successful interactions with our environment may induce a general belief that our perceptual capabilities are dependable. After all, such capabilities allow us to carry out survival activities (e.g., procuring food, reproduction, shelter building, defense, etc.). Nevertheless, our mechanisms for fast and intuitive judgments may be unsuitable for the visual complexities of our modern world (Chabris & Simons, 2009).

In Experiment 1, participants were informed about the subtle and the salient changes in the Princess Card Trick and they made offline predictions about the likelihood that other people would detect them. Participants predicted that others would detect the salient change more easily than the subtle change. These predictions were based on beliefs about intrinsic cues, and they were consistent with the actual detection rates found in Experiment 2. These findings dovetail results from a study examining the impact of scene complexity on change detection, which found that participants accurately predicted lower change detection as the number of objects in the scene increased (Beck et al., 2007). However, when participants watched the magic trick without prior knowledge about the visual change (Experiment 2), predictions varied as a function of the experience cue (change blindness and change detection). Specifically, experiencing change blindness led to lower predicted detection rates regardless of the type of change. These findings concur with Ortega et al., (2018), who found egocentric biases in predictions that were related to a range of magic tricks that were based on inattentional blindness and change blindness. This bias has also been reported in nonvisual domains (e.g., Kelley & Jacoby, 1996; Koriat & Ackerman, 2010). Interestingly, even though most participants were able to detect the salient change, those who experienced change blindness made significantly lower predictions for others’ ability to detect it. This finding suggests that experience cues are more impactful than intrinsic cues when making online metacognitive judgments. Further support for this claim is provided by the comparison between online and offline predictions.

Online judgments made by participants who experienced change blindness were significantly lower than offline judgments for both types of change. This is consistent with the influence of System 1 over metacognitive judgments about others. Thus, participants made judgments on the basis of feelings derived from their experience rather than taking into account the characteristics of the stimuli as in Experiment 1. Interestingly, online predictions made by participants who detected the change were similar to offline predictions. This finding is consistent with the confirmatory bias of System 1: Seeking for confirming evidence is less effortful than testing prior beliefs. Thus, people tend to pay more attention to information that confirms their beliefs and may exaggerate the likelihood of improbable occurrences (Kahneman, 2011).

Finally, we examined whether the experience cue would also influence predictions for a nonexperienced change of differing saliency. Contrary to what we hypothesized, the experience cue had no influence, whereas the intrinsic cue produced significant differences: Similar to findings from Experiment 1, participants predicted a higher detection rate for the salient change than for the subtle change. In this case, System 2 may have overridden the output of System 1. On the other hand, this result may point to a limitation of transfer: Our lived experience in one salience context cannot easily be transferred to a new simulated context, leading to an overreliance on those features that *can* be easily simulated (e.g., saliency).

The present study provides evidence for the role of experience in belief updating. In the verbal memory context, failure to remember will trigger efforts to retrieve the information or relearn the material (Kornell & Vaughn, 2016; Kornell et al., 2009), making apparent the limits of memory. In contrast, instances where we have failed to detect a change are rarely available to us, giving us little opportunity to update our beliefs about visual awareness. As a result, we assume that we can consciously perceive objects and changes that in reality are hard to notice. Demonstrations of metacognitive errors could be used to educate people about the counterintuitive nature of perceptual limitations, which do not trigger any red flags that would allow us to update incorrect beliefs. Indeed, an important message is “be wary of your intuitions, especially intuitions about how your own mind works” (Chabris & Simons, 2009, p. 241).

Overall, the results of this study point toward joint contributions of experience and information cues to metacognitive judgments about other people’s change detection abilities. Understanding how visual metacognition might be influenced by different types of cues has practical implications. For instance, cognitive ergonomics focuses on how cognition affects work and vice versa (Hollnagel, 1997). Software developers could rely on their own experience to design programs. However, as we have demonstrated, reliance on prior experience could potentially lead to biases and thus to poor user experience. On the other hand, software developers could rely on information cues that better fit the users’ cognitive capabilities.

Demonstrations of change blindness contradict the belief that our perceptual capabilities are dependable, but successful interactions with our environment seem to support such a belief. However, people might not be able to verbalize their beliefs in a precise way (Cohen, 2002). Epistemic feelings such as surprise carry information about mental states, thus serving a metacognitive function by implicitly representing the beliefs of cognitive agents (Arango-Muñoz, 2014). People often experience surprise when expectations are violated (Dennett, 2002), such as in magic tricks (Danek et al., 2015; Parris et al., 2009). Future studies might address the role of surprise in understanding visual metacognition. Moreover, further research might elucidate the role of System 1 and System 2 in overestimation and underestimation errors in judgments about others’ perceptual abilities.

## Appendix A: Magic Trick Description

Read the following description carefully: In a magic trick, the magician shows five cards and asks you to choose one and think of it intensely. The magician says that she will try to read your mind from a distance to find out what your card is. Finally, she shows that she has made a card disappear, she shows the four remaining cards and you realize that the card that you chose disappeared. To accomplish this trick, the magician secretly changes the cards. Thus, the cards shown at the beginning (Figure 1A or 1C in this document) are different from the cards shown at the end (Figure 1B or 1D in this document). Look at the figures and notice the difference between the two. Then click “Next” to watch the trick.

## Appendix B: Questionnaire

1. Have you watched this magic trick before? (Yes/No)
2. Did you notice anything unusual about the trick? (Yes/No) If yes, please describe.
3. When you watched the video, did you notice that the cards shown at the end [Figure 1B or 1D in this document] were different from the cards displayed at the beginning [Figure 1A or 1C in this document]? (Yes/No)
4. How many people out of 10 do you believe would notice that the cards change when watching the trick? In another version of the same magic trick the cards change from [Figure 1A to 1B or Figure 1C to 1D in this document]. Notice the difference between the two figures. Then click “Next” to see the trick.
5. When you watched the video, did you notice that the cards shown at the end were different from the cards displayed at the beginning? (Yes/No)
6. How many people out of 10 do you believe would notice that the cards change when watching the trick?
